# An Evaluation of a Continuing Education Program for Family Caregivers of Ventilator-Dependent Children with Spinal Muscular Atrophy (SMA)

**DOI:** 10.3390/children4050033

**Published:** 2017-04-29

**Authors:** Deborah S. Boroughs

**Affiliations:** Special Program Development, BAYADA Home Health Care Pediatric Specialty Practice, 5 Terri Lane, Suite 11, Burlington, NJ 08016, USA; dboroughs@bayada.com; Tel.: +1-610-742-1972

**Keywords:** pediatric ventilator dependence, spinal muscular atrophy, medically-complex children, complex home care, family caregivers

## Abstract

Until 25 years ago, there were limited options for long-term mechanical ventilation of children, and the majority of children were cared for in hospitals. However, with improving technology, the pediatric intensive care unit has moved from the hospital to a home setting, as children with increasingly complex healthcare needs are now often cared for by family members. One of the most complex care conditions involves ventilator and tracheostomy support. Advanced respiratory technologies that augment natural respiratory function prolong the lives of children with respiratory compromise; however, this care often comes with serious risks, including respiratory muscle impairment, respiratory failure, and chronic pulmonary disease. Both non-invasive assisted ventilation and assisted ventilation via tracheostomy can prolong survival into adulthood in many cases; however, mechanical ventilation in the home is a high-stakes, high risk intervention. Increasing complexity of care over time requires perpetual skill training of family caregivers that is delivered and supported by professional caregivers; yet, opportunities for additional training outside of the hospital rarely exist. Recent data has confirmed that repetitive caregiver education is essential for retention of memory and skills in adult learners. This study analyzes the use of continued education and training in the community for family caregivers of ventilator-dependent children diagnosed with spinal muscular atrophy (SMA).

## 1. Introduction

It is estimated that 4800 children living in the USA are currently supported by mechanical ventilation at home, and the prevalence of home mechanical ventilation for children is also similar in other developed nations [1]. Many non-elective hospital readmissions of these mechanically-ventilated children are typically avoidable and are often related to inappropriate caregiver interventions [2].

Spinal muscular atrophy (SMA) is a disease that affects the motor nerve cells in the spinal cord, resulting in an inability to walk, eat, or breathe, and is the number one genetic cause of death for infants. SMA is caused by a mutation in the survival motor neuron gene 1 (*SMN1*). Without respiratory support, 90% of children diagnosed with SMA type 1 die before 12 months of age and 100% die by 24 months of age [3]. The most serious dangers of SMA are respiratory muscle impairment, respiratory failure, and chronic pulmonary disease. Long-term survival depends upon the choice of treatment. Advanced respiratory technologies that augment natural respiratory function prolong the lives of children diagnosed with SMA types 1 and 2. Both non-invasive assisted ventilation and assisted ventilation via tracheostomy can prolong survival to over 20 years of age in many cases. Family members of children with SMA types 1 and 2 who opt for mechanically assisted ventilation, typically become the primary caregivers for their children at home. Mechanical ventilation in the home is a high-stakes, high risk intervention. As neurodegenerative changes associated with SMA occur over time, the increasing complexity of care requires updated skill training of family caregivers that is delivered and supported by professional caregivers. In the USA, over the past 20 years, caregiver error has resulted in a consistent 18–20% of the mortality of all ventilator-dependent children cared for at home [4].

Formal clinical teaching for family caregivers typically ends at the time of discharge from the hospital, and opportunities for additional skills training outside of the hospital setting rarely exist. Thus, a gap in education and support may develop at-home when initial hospital training for the family caregivers does not keep pace with at-home clinical changes. The demands of round-the-clock care by family caregivers allow little opportunity for families to travel to tertiary centers to receive additional training or to practice emergency response, therefore access to evidence-based practice changes, protocols and technological advances remains limited. Family caregivers may not encounter an emergency for years after a child’s initial discharge from the hospital and they often have difficulty recalling how to respond quickly and successfully in the midst of an emergency. Anticipating and preparing for future needs are crucial to successful respiratory management of a child with SMA in the home. In order for family caregivers to respond to the complex needs of their children, they must be provided with convenient, repetitive training opportunities either in their own home or at a nearby community simulation training center [5]. Portable electronic simulation training provided in a local simulation lab or at home offers convenient practice opportunities to replicate and prepare for real-life emergencies and to introduce new skills or improved techniques.

Research has confirmed that repetitive caregiver education improves survival rates, decreases frequency and lengths of hospital stays, and decreases complications and costs for these children [1]. Recognizing this need, Bayada Home Health Care Pediatric Specialty Practice (Burlington, NJ, USA) created a community-based training curriculum to bridge the knowledge gap for family caregivers of children diagnosed with SMA. Therefore, this study was created to evaluate the effectiveness of this training.

## 2. Materials and Methods

Participants for the project were recruited from the organization’s SMA client database. The families received their testing and training in a simulation lab at the nursing agency, which provides their homecare nurses. The design of the nursing agency simulation labs was created to represent a child’s bedroom, including a Gaumard (Miami, Florida USA) high-fidelity or Laerdal (Wappingers Falls, NY, USA) mid fidelity manikin in the cribs with all of the necessary equipment and supplies for the scenarios at bedside (e.g., mechanical ventilators and Bilevel Positive Airway Pressure (BiPAP) machines. The trainers are nurse simulation educators who are registered nurses that have completed simulation training by the simulation equipment vendor and by internal simulation educators. During the training sessions of this project, the simulation educators either stood behind a room divider to observe the caregivers remotely on a computer screen or stood directly next to the caregivers to provide direct instruction, and all training sessions were videotaped. The sessions in the lab averaged four hours in length, and additional coaching was provided as needed. In-home visits were then made by each family’s clinical manager at three months post-training, for a session that lasted approximately two hours, in which the clinical managers assessed the skill level and technique of the family caregiver as the caregiver provided care, including a tracheostomy tube change and pulmonary toilet, for the child at bedside.

The entire training curriculum was based on an eight-step program: (1) an untimed pre-test for family caregivers to assess their baseline knowledge of emergency response during the scenarios they were about to perform. Two tests were developed, one for those children receiving non-invasive ventilation (BiPAP) and one for those receiving mechanical ventilation via a tracheostomy; (2) in the electronic simulation lab, the family caregivers were asked to perform responses to simulated emergency scenarios without coaching by the simulation nurse educator; (3) debriefing of emergency scenario responses and remediation using correct technique, which were taught by the simulation nurse educator; (4) the initial scenarios were repeated by the caregivers using correct technique and sequence without prompting by the nurse educator; (5) untimed post-tests were administered; (6) a satisfaction survey regarding the training was completed by each participant; (7) 3-month post-training visits and skill assessments of the family caregivers in their home at the bedside of the child, which were performed by each family’s clinical nurse manager; (8) 6-month post-training surveys via telephone by the project manager, which were obtained to glean anecdotal information and the level of satisfaction from the participants.

## 3. Results

The sample included 11 children aged between six months to 18 years (Table 1). They resided in seven states and received primary medical care at five different children’s hospitals. Family caregivers who participated were the children’s primary caregiver at home and included parents, grandparents and foster parents. Participating family caregivers had educational levels that ranged from high school graduate to college degrees, and therefore all training materials were designed with simplistic, clear language, to accommodate all levels of education. Additionally, multiple ethnicities were represented among the 11 families. Caregivers traveled to the electronic simulation lab located at a BAYADA nursing agency closest to their homes.

As previously mentioned, the curriculum included: untimed pre-testing; un-coached performance of response to simulated emergency scenarios; debriefing and remediation; repeat of scenarios using correct technique and sequence; untimed post-test; satisfaction survey; a 3-month post-training home visit and skill assessment of the family caregivers by a clinical manager at the child’s bedside; and a 6-month post-training phone survey by the project manager. Scenarios included the primary emergencies that occur at home (Table 2), with training conducted over a period of six months. The Framework Method of Analysis—a systematic yet flexible analysis—was used to measure the qualitative data gleaned from the study and to produce structured outputs of summarized information [6]. Three and six-month post-training surveys of the family caregiver participants added anecdotal insights.

An independent analyst reviewed all of the videotaped training sessions of the family caregivers for the project and collected the following data provided in the sections below, with the final results listed in Table 3.

### 3.1. Transcription and Familiarization

A good quality audio recording of each session was obtained. The content is of primary interest and provided a good opportunity to review the data. Using the audio recording was vital for interpretation, as analytical notes and impressions were able to be accurately collected.

### 3.2. Coding: Creating an Analytical Framework

Coding was done to classify the data, so that comparisons between the individual recordings can be made. As caregivers had varying degrees of skill and confidence, both pre- and post-testing, and instructors had unique styles of presenting the standardized teaching, coding allowed for a more holistic impression. The codes were then grouped into defined categories of the training, primarily the prescribed scenarios and the trainee responses.

The data was condensed into three themes: (1) nurse trainers; (2) family caregivers and (3) clinical nurse managers. As the qualitative data was voluminous and accounted for more than 50 h of videotaping, in charting the data, a balance was achieved by reducing the data to common themes and retaining the individuality of the trainee caregivers.

### 3.3. Interpreting the Data

Overall, the training was found to be highly beneficial for all participants (Table 3). One hundred percent of the participants reported a greater level of confidence in their ability to respond effectively to emergencies. Three-month post training evaluation by clinical nurse managers in client homes of caregivers with their own children confirmed this. Several parents reported a desire for future training to be done in their homes rather than in a simulation lab, for convenience and for the opportunity to have more “real life” practice at the bedside. Additionally, during the six-month post-training phase of the project, 36% (4/11) of the children were hospitalized for infections and/or respiratory distress; however, each family caregiver, based on the training they received, recognized the immediate need for medical intervention to prevent respiratory illness from progressing to a higher level of acuity. Several reported that they were able to respond to tracheostomy emergencies at home without having to go to the emergency room and attributed their successful emergency response to their recent preparation in the simulation lab.

It was anticipated that on-going and convenient family caregiver training that utilizes electronic simulation would provide a stress-free learning experience with no risk to children and would empower the caregivers with a greater mastery of skills and therefore a greater confidence that results in improved clinical outcomes and fewer hospitalizations for their children. Some anecdotal responses by the family caregivers at the conclusion of this study are provided in Table 4, and serve as convincing arguments as to the satisfaction levels of caregivers for this training program.

Additionally, some difficulties did arise, which mostly stemmed from the following issues, and should be considered in future studies: (1) some participants had difficulty retaining a large amount of knowledge that is delivered in a single session; (2) there may, in fact, be too many aspects of care to learn in a single session; (3) the skills being taught were quite complex; (4) some participants appeared to be in a “panic mode” that may have impeded learning; (5) there may not have been sufficient time to practice the new skills.

## 4. Discussion

Most parents or guardians of children with complex healthcare needs become the primary caregivers at home. They recognize the need for continued education and desire practice opportunities at the nursing office simulation lab and at home after they are discharged from the hospital where they received their initial skills training. Although insurance benefits vary among states, ventilator-dependent children typically receive between eight and 16 h per day of professional nursing care in the home. The remaining hours of care are provided by family caregivers without assistance. Despite technological advances, particularly with monitoring systems for children supported by mechanical ventilation at home, the incidence of preventable deaths remains constant over the past 20 years at 18–20% of all reported deaths [4], and the primary cause is caregiver error [2,4]. In order to decrease the number of preventable deaths at home, updated skills training of the family caregivers is needed, which is delivered and supported by professional caregivers, and must also keep pace with the increasing complexity of care for these children [1].

One limitation of the study is that there is not a comparison group and the numbers are small for determining with certainty that outcomes were better for those caregivers who participated in the training program. This would require major funding and a multicenter trial, among other obstacles, as well as potential ethical concerns. However, this study was instead meant to target the well-known knowledge of adult learning, the deterioration of skills not routinely utilized over time, and the importance of repetition for memory retention, and thusly decrease the gap in learning for caregivers through a targeted curriculum of training.

## 5. Conclusions

While small, this study serves as a first step to indicate the need for homecare professionals to provide on-going simulation training and practice opportunities to family caregivers of mechanically ventilated children with complex diagnoses. In search of a continuing decrease in the knowledge gap of caregiver training, BAYADA Home Health Care Pediatric Specialty Practice has expanded this study to provide on-going training and emergency response practice to family caregivers of children with other complex medical needs at a community simulation center or in the patient’s home at bedside. This project met the objectives of preparing and empowering family caregivers with skill mastery, greater confidence, and improved clinical outcomes for their children. Most importantly, this study highlights that continuing education may lead to better clinical outcomes at home, fewer emergency room visits, fewer hospital readmissions, a potential reduction in associated costs, and a better overall quality of care for children with complex healthcare needs.

## Figures and Tables

**Table 1:** Demographics of sample children (*n* = 11) with spinal muscular atrophy (SMA).

Client	Caregiver	Ventilation	SIM Lab Date	3-Month Survey Date	6-Month Survey Date	Client Age	SMA Type
O.H.	C.M.	BiPAP	9 April 2015	9 July 2015	9 October 2015	5 years	2
K.C.	D.C.	Trach/Vent	16 April 2015	16 July 2015	16 October 2015	6 years	1
M.S.	T.F.	BiPAP	20 April 2015	20 July 2015	20 October 2015	6 years	2
T.B.	K.B.	Trach/Vent	12 March 2015	12 June 2015	12 September 2015	1.5 years	1
T.M.	B & T.M.	Trach/Vent	20 March 2015	20 June 2015	20 September 2015	4 years	2
A.R.	M.R.	Trach/Vent	24 April 2015	24 July 2015	24 October 2015	6 months	1
J.A.	T.P.	BiPAP	15 April 2015	15 July 2015	15 October 2015	7 years	2
J.F.	A.F.	BiPAP	7 April 2015	7 July 2015	7 October 2015	16 years	1
M.F.	L & L.F.	BiPAP	3 April 2015	3 July 2015	3 October 2015	18 years	1
J.R.	C.R.	Trach/Vent	18 March 2015	18 June 2015	18 September 2015	4 years	1
J.C.	D.R.	BiPAP	27 April 2105	27 July 2015	27 October 2015	6 years	1

BiPAP: Bilevel Positive Airway Pressure; Trach/Vent: tracheostomy and ventilator.

**Table 2:** Emergency scenarios for BiPAP and mechanical ventilation via a tracheostomy.

Non-Invasive Mechanical Ventilation (BiPAP) Scenarios	Mechanical Ventilation Via Tracheostomy Scenarios
Agitation of child while on BiPAP	Decannulation with inability to re-establish airway
Respiratory distress while on BiPAP	Ventilator malfunction
Power outage response	Respiratory distress response
CPR for the child on BiPAP *	Power outage response
	CPR for the child with a tracheostomy

* The American Heart Association (AHA) 2010 guidelines were used for cardiopulmonary resuscitation (CPR) training. (The new 2015 AHA guidelines were not released prior to the study.) For resuscitation of a child with a tracheostomy, the educator removes the face mask from a Bag-Valve-Mask (BVM) resuscitation bag and attaches the resuscitation bag directly to the tracheostomy tube port. The family caregivers are instructed to perform CPR as they were taught in the hospital prior to discharge. Hospital teaching is reinforced throughout the simulation lab sessions.

**Table 3:** Test and survey interpretations.

Results	All but one caregiver improved pretest scores on post-tests.
	All caregivers reported that they received new information.
	All caregivers reported satisfaction or high satisfaction with the training techniques.
	All caregivers reported that the nurse simulation educators were knowledgeable.
	All caregivers reported increased confidence in care giving as a result of the training.
	All caregivers reported feeling better prepared to recognize and handle emergencies as a result of the training.
	All caregivers reported that the emergency training would be valuable to caregivers of children with other complex medical needs that require mechanical ventilation at home.
	All caregivers evaluated by clinical nurse managers in their homes at the child’s bedside were determined to be proficient in caregiver skills and techniques for their children.
Recommendations for future training	Create identical checklists for both nurses and family caregivers to follow step-by-step during training.
	Have a member of the training team assume responsibility for reviewing the videos and report the differences between planned-to-do and real-world to-do to the team.

**Table 4:** Family caregiver responses at post-training.

*“[My son] developed a respiratory infection that rapidly progressed. I was having difficulty clearing his airway and keeping his oxygen saturation within his normal limits. I immediately thought about the training I received in the sim lab. I remained calm and began the stepwise approach to assessment of his condition I had learned in the simulation lab. I recognized that he was heading rapidly from respiratory distress toward respiratory arrest. I called 911 and the children’s hospital. The children’s hospital airlifted my son by helicopter directly to the hospital. By the time we reached the hospital by car, my son was in the operating room having a tracheostomy tube placed. He is now receiving mechanical ventilation with a tracheostomy and a ventilator. He has been in the hospital for more than two months while we receive new caregiver training for invasive ventilation and while he recovers. The sim lab training led to my accurate recognition of a dire emergency and I was able to respond calmly and effectively which saved his life. We look forward to future training in the BAYADA sim lab to reinforce and refresh the skills we are learning now in the hospital. I believe all parents who have children with complex respiratory needs should be offered the emergency training we received. It isn’t until an emergency occurs that you realize how important it is to respond quickly and effectively.”*
*“The training is awesome and is important for all parents of children like mine.”*
*“In the future, if my child receives new orders or a new piece of equipment, I would like to receive additional training.”*
*“After the training, [my child] could no longer be adequately supported by BiPAP. He received a tracheostomy and is now supported by [invasive] mechanical ventilation. I will want new emergency preparedness training in the near future.”*
*“At first the training was nerve-wracking. The simulation educator soon put me at ease, and I was able to learn new information. My confidence in caring for my child was boosted by the training.”*
*“I am a seasoned caregiver, but the practice was great and I’m glad to know that I will be able to recognize and handle a respiratory emergency.”*
*“The training was very, very helpful.”*
*“I feel like I can better anticipate emergencies.”*
